# An Enhanced CT-based Radiomics Model for Predicting the Anaplastic Lymphoma Kinase Mutation Status in Lung Adenocarcinoma

**DOI:** 10.2174/0115734056376470250718053430

**Published:** 2025-07-31

**Authors:** Zaixian Zhang, Taijuan Zhang, Hui Ding, Shunli Liu, Zhiming Li, Yaqiong Ge, Lei Yang

**Affiliations:** 1 Department of Radiology, The Affiliated Hospital of Qingdao University, Qingdao, China; 2 Department of Radiology, Qingdao Women and Children's Hospital, Qingdao, China; 3 Department of Radiology, Qingdao Hiser Hospital Affiliated of Qingdao University (Qingdao Traditional Chinese Medicine Hospital), Qingdao, China; 4 GE Healthcare China, Shanghai, China

**Keywords:** Lung neoplasms, Anaplastic lymphoma kinase, Radiomics, X-ray, CT, ALK mutations

## Abstract

**Introduction:**

This study aimed to explore the relationship between radiomics features and anaplastic lymphoma kinase (ALK) gene mutation status in lung adenocarcinoma and to develop a radiomics nomogram for preoperative prediction of ALK mutations.

**Methods:**

A retrospective analysis was conducted on 210 patients with histologically confirmed lung adenocarcinoma (50 ALK mutation-positive, 160 mutation-negative), divided into training (n=147) and validation (n=63) cohorts (7:3 ratio). Preoperative enhanced CT images were analyzed using ITK-SNAP for region-of-interest delineation, and radiomics features were extracted *via* A.K. software. The least absolute shrinkage and selection operator algorithm selected features to generate a radiomics score. Multivariate logistic regression identified independent risk factors, and a radiomics nomogram combining clinical features and radiomics signatures was developed. Model performance was evaluated using AUC in both training and validation sets.

**Results:**

Nineteen radiomics features were selected to construct the radiomics signature. The signature achieved an AUC of 0.89 (95% CI: 0.84–0.95) in the training set and 0.79 (95% CI: 0.63–0.95) in the validation set. The radiomics nomogram demonstrated superior performance (AUC=0.80, 95% CI: 0.63–0.97) compared to the clinical model alone (AUC=0.66, 95% CI: 0.47–0.85) in the validation set. While the nomogram showed no statistically significant improvement over the radiomics signature alone (*P*>0.05), it outperformed the clinical model significantly (*P*<0.001 in training; *P*=0.0337 in validation).

**Discussion:**

The radiomics nomogram integrating clinical and radiomics data demonstrated robust predictive capability for ALK mutations, highlighting the potential of non-invasive CT-based radiomics in guiding personalized treatment. However, the lack of significant difference between the nomogram and radiomics signature alone suggests limited incremental value from clinical variables in this cohort. Limitations include the retrospective design, single-center data, and class imbalance (fewer ALK-positive cases), which may affect generalizability. External validation is warranted to confirm clinical utility.

**Conclusion:**

The CT-derived radiomics signature and nomogram show promise for preoperative ALK mutation prediction in lung adenocarcinoma. These tools could enhance clinical decision-making by identifying candidates for targeted therapies, though further validation is needed to optimize their application in diverse populations.

## 1 INTRODUCTION

Lung cancer remains the most common and predominant cause of malignancy-associated mortality around the world [1]. The diagnosis is usually made at a locally advanced or metastatic stage, leading to a five-year survival rate of less than 25%. Lung adenocarcinoma has emerged as the most prevalent subtype of lung cancer, exhibiting the highest incidence of gene mutations [2, 3]. Recently, the identification of driver genes in cancer and their implications in predicting the efficacy of targeted therapies have significantly contributed to the development of diagnostic and therapeutic approaches for advanced lung adenocarcinoma. In particular, anaplastic lymphoma kinase (ALK) mutations are present in roughly 3–5% of lung cancer patients, higher in patients with lung adenocarcinoma. They are now the second most common molecular changes in lung cancer treatment, after epidermal growth factor receptor (EGFR) mutations [4-6]. Therefore, identifying ALK rearrangements in patients with lung adenocarcinoma is essential for the treatment strategy.

Tumor tissue biopsy is still the gold standard for mutational research, but it is costly, time-consuming, unfeasible for repeated testing, and unable to assess tumor heterogeneity. Furthermore, biopsy is invasive and causes discomfort to patients, which results in many patients missing out on treatment options and possibilities. Consequently, there is a need for a non-invasive, convenient, and more reliable method to evaluate the entire tumor for detecting the mutation status. Computed tomography (CT) is widely used as a modality for lung cancer screening and diagnosis due to its non-invasive nature and repeatability for monitoring patient conditions. Previous studies have demonstrated some CT imaging characteristics associated with ALK gene rearrangements in lung cancer, such as central tumor location, irregular margins, homogeneous texture, pleural involvement, and distant metastases [7-10]. However, the assessment of these conventional CT imaging features is highly dependent on the radiologist's expertise and can be time-consuming. Radiomics, an emerging field in medical image analysis, has gained significant attention due to its cost-effectiveness and ability to quantify tumor heterogeneity. It has enabled improved assessment of treatment response and prediction of molecular pathways [11, 12]. Radiomics quantifies the distribution and relationship of pixel intensities in CT images, revealing subtle differences that may not be discernible to the human eye. This overcomes the limitations of conventional CT imaging. Radiomics enables the conversion of medical images into quantitative data, which can be analyzed to predict the clinical stage, histopathological diagnosis, genetic mutation status, and prognosis of lung cancer patients. Additionally, it can assess treatment efficacy [13-16]. In recent years, numerous studies have explored the use of CT image-based radiomic features to predict the EGFR gene mutation phenotype in lung adenocarcinoma [17-19]. For instance, features extracted from CT images, such as size, margin characteristics, texture, and homogeneity, have been used to identify EGFR mutation status. However, only a few studies have explored ALK mutation prediction in lung adenocarcinoma patients using CT image-based radiomics [20-22], probably primarily due to the low prevalence of ALK mutation-positive (ALK Mut) lung cancer [23]. The study findings [20-22] demonstrated that combining CT image-based radiomic features with clinical characteristics enhances the identification of ALK mutations in lung adenocarcinoma. The imaging characteristics of ALK Mut lung tumors and their relationship to molecular phenotypes are less well-understood compared to those of EGFR mutation-positive tumors, and further studies utilizing larger datasets and prospective designs are needed.

Therefore, we aimed to develop and validate an enhanced CT radiomics-based model for diagnosing ALK rearrangement status in lung adenocarcinoma and to evaluate whether incorporating conventional CT characteristics and clinical data enhances the predictive performance of the model.

## 2 MATERIALS AND METHODS

### 2.1 Patients

The Institutional Review Board waived the requirement for informed consent, and the local ethics committee authorized the protocol.

From April 2018 to September 2020, a total of 2240 patients with lung adenocarcinoma at our institution who were confirmed by surgery or biopsy were identified as the subjects of this retrospective study. The inclusion criteria were as follows: (1) a pathological confirmation of primary lung adenocarcinoma through histological examination; (2) availability of ALK mutation gene detection results; (3) availability of dual-phase contrast-enhanced CT scans prior to treatment initiation; and (4) solid tumors without a ground-glass component and larger than 10 mm in size. The exclusion criteria were: (1) incomplete clinical or imaging data (n =93); (2) CT images with a poor image quality (n = 36); (3) patients who received previous treatment or invasive surgery before CT scans (n = 225); and (4) a history of other primary malignancies (n = 108). All cases were divided into ALK Mut or ALK mutation-negative (ALK WT) according to the results of genetic testing. Accordingly, 627 patients met the eligibility criteria for the study. A total of 50 ALK Mut patients were finally identified as the study group. Meanwhile, ALK WT adenocarcinoma patients (n = 160) were randomly selected from the enrolled cases at a ratio of approximately 1:3 to serve as the control group. Fig. (**1**) presents the patient selection flowchart. Therefore, a total of 210 cases were collected, with the age range of the included patients being 30–81 years, a mean age of 60.1 ± 9.4 years, including 88 males and 122 females. Clinical data, including sex, age, smoking history, clinical stage, maximum tumor diameter, the location of the tumor and metastasis, were extracted from the hospital information system.

### 2.2 CT Protocol

Chest dynamic contrast-enhanced CT scans were performed using a multi-detector row scanner (Brilliance iCT 256, Philips Healthcare; or SOMATOM Definition Flash, Siemens Medical Systems), with a scanning range from the lung apex to the costophrenic angle. The scanning parameters were as follows: tube voltage 120 kV, automatic tube current modulation technique, matrix 512 × 512, slice thickness 5 mm, reconstructed slice thickness 1 mm, slice spacing 1 mm, and pitch of 1.375–1. During the CT scan, the patients were instructed to hold their breath. Contrast agent (ioversol, containing 370 g iodine/L) of 100 mL was injected *via* a Medrad Stellant dual-syringe high-pressure injector at an injection rate of 3.5 mL/s. A Medrad Stellant dual-syringe high-pressure injector was used to inject 100 mL of the contrast agent (ioversol, which contains 370 g iodine/L) at a rate of 3.5 mL/s. At 25 and 70 seconds following the start of the contrast agent injection, the arterial phase and venous phase CT scans were obtained, respectively.

### 2.3 Image Acquisition and Segmentation

Chest dynamic contrast-enhanced biphasic DICOM format images of 210 patients were retrieved and exported from our hospital's picture archiving and communication system (PACS). The window width and window level of the axial images were uniformly processed at the level of the largest cross-section of the lesion in the arterial and venous phases (mediastinal window, with a window width of 400 HU and a window level of 40 HU). Two thoracic radiologists (with 10 and 15 years of experience, respectively) independently reviewed and interpreted transverse reconstructed CT images, blinded to the patients' clinical data. The radiologists evaluated radiological features derived from subjective CT interpretation and reached a consensus agreement on their findings. Then, ITK-SNAP 3.8 software (http://www.itksnap.org, USA) was used to manually delineate the regions of interest (ROIs) along the lesion contours, avoiding areas such as blood vessels, pleura, or other soft tissues as much as possible. The radiologists performed manual 2D ROI segmentation of the arterial and venous phase images. To assess feature extraction reproducibility, 30 CT images were randomly selected to calculate inter- and intra-class correlation coefficients (ICCs). Good consistency is indicated by an ICC value greater than 0.75.

### 2.4 Features Extraction

Prior to feature extraction, the original images underwent normalization. A.K. software (Analysis ToolKit 1.0.3; GE Healthcare, China) was used to automatically extract 396 quantitative texture features from the ROIs outlined in the enhanced CT images, including the following categories of radiomic features: histogram, form factors, Haralick features, gray-level co-occurrence matrix (GLCM) features, gray-level size zone matrix (GLSZM) features, and gray-level run length matrix (GLRLM) features. The arterial phase and venous phase CT scans were subjected to separate image analysis and feature extraction procedures using the same technique. As a result, 112 arterial phase features and 87 venous phase features were extracted.

### 2.5 Features Selection and Radiomics model Construction

In a 7:3 ratio, patients were randomized to either the training set (n = 147) or the validation set (n = 63). In order to remove redundant and unnecessary features from the training set, the most valuable features were chosen using the minimum redundancy maximum relevance (mRMR) method. The top 30 features with little redundancy and high relevance were kept for the selection procedure. Then, the least absolute shrinkage and selection operator (LASSO) regression method was employed to further retain the most relevant features. Using 10-fold cross-validation, the lambda(λ) value that minimized the model error was chosen to optimize the LASSO regression model. By excluding radiomic features with zero coefficients and keeping only those with non-zero coefficients, the radiomics scores were calculated for each patient using the linear combination approach of these features and their corresponding coefficients. We evaluated the predictive accuracy of radiomics scores by assessing the area under the receiver operating characteristic (ROC) curve (AUC) using training and validation datasets.

In order to determine potential risk factors for predicting ALK mutation, univariate logistic regression analysis was conducted on features with *P*<0.1 after a preliminary examination of the clinical predictive features of ALK mutation. To build the multivariable model for differentiating between lung adenocarcinoma and ALK mutation status, multivariate logistic regression analysis was used to check for independent predictors and the best radiomics scores. Using a likelihood ratio test and Akaike's information criterion as the termination criterion, logistic regression used backward stepwise selection. On the basis of the training set's screened features, a nomogram model was built.

### 2.6 Model Evaluation and Validation

The diagnostic value of the radiomic nomogram was identified, calibrated and evaluated in the training and validation sets. The Hosmer-Lemeshow test was used to analyze the difference after the calibration curve was plotted to determine the radiomic nomogram's goodness of fit. The discrimination performance of the radiomic nomogram was quantified by the ROC curve and AUC. The ROC discrepancies between the various models were assessed using the DeLong test. The decision curve analysis (DCA) was utilized to determine the net benefit of a number of threshold probabilities in the training and validation sets in order to assess if the nomogram was sufficiently robust for clinical application. Fig. (**2**) displays the workflow for data analysis.

### 2.7 Statistical Analyses

For statistical analysis, we utilized R statistical software (v.3.2.1; https://www.Rproject.org) and SPSS software (https://www.ibm.com, version 26.0). Continuous variable differences were examined using the Wilcoxon's test or the independent t-test. The Fisher's or Chi-square tests were used to assess categorical variables. The diagnostic performance of multivariate models was assessed using ROC analysis and AUC. A value of *P* < 0.05 was deemed statistically significant.

## 3 RESULTS

### 3.1 Clinical Characteristics of the Study Subjects

A total of 210 patients participated in the study. According to Table **1**, there was a statistically significant difference in age, smoking history, TNM stage and metastasis between ALK Mut and ALK WT patients (*P* < 0.05), and there were no statistically significant differences observed in sex, maximum diameter and location (*P* > 0.05).

### 3.2 Feature Selection and Construction of the Radiomics Model

**Fig. (3):**
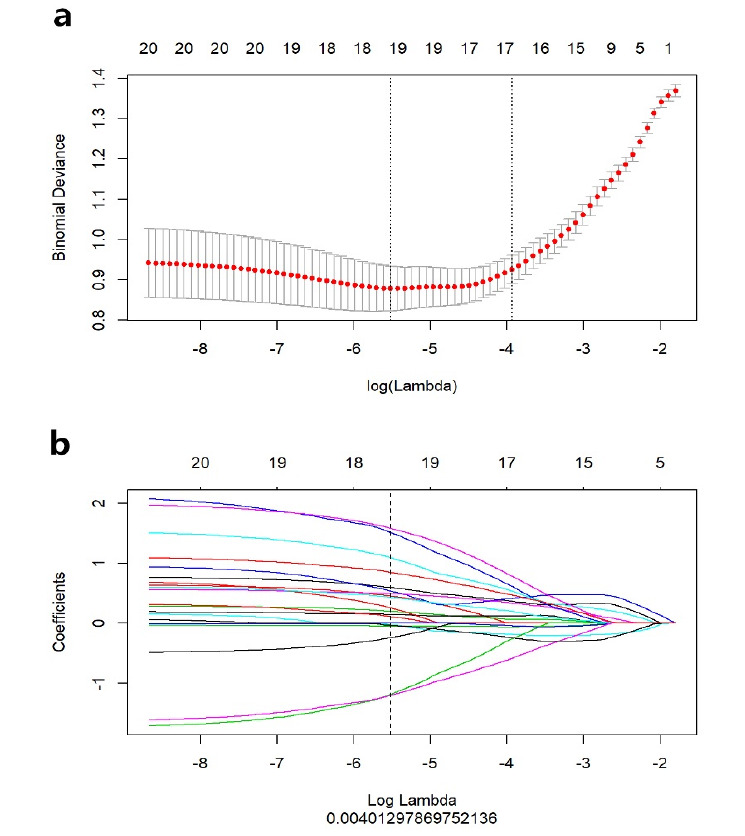
Image of LASSO 10-fold cross-validation plot. (**a**) The selection of the regularization parameter λ in the LASSO model. The red dot represented the average bias value of the model corresponding to different λ values. The optimal λ value was the abscissa corresponding to the lowest point of the model bias curve. (**b**) The color line represented the change curve of the coefficient of the feature with the value of λ. The dashed line corresponds to the optimal value of λ. 19 features with non-zero coefficients were retained.

**Fig. (4):**
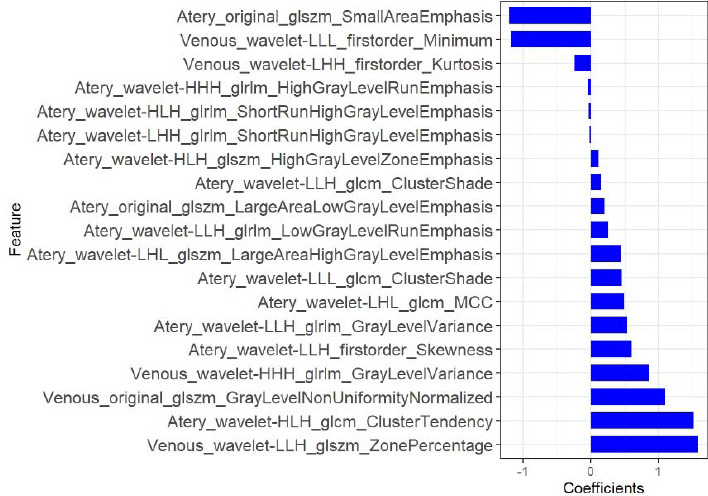
LASSO Regression Analysis with 19 Retained Features. The y-axis lists the 19 features with non-zero coefficients when the optimal λ value was selected. The x-axis showed the corresponding coefficients in the LASSO regression model. Features with larger coefficients had higher predictive value.

As described in the Materials and Methods section, mRMR and LASSO were applied for feature selection, and according to the optimal λ value (Fig. **3a,b**), 19 features were finally selected (Fig. **4**). Among them, 14 features came from the arterial phase and 5 features from the venous phase. Each patient's radiomics score was determined using the 19 features that were chosen and the associated coefficients, and then the radiomics scores of the ALK Mut and ALK WT groups in the training set and validation set were compared, respectively. The Wilcoxon test was used to assess the differences between the two groups. Fig. (**5a,b**) shows that the classification difference between the two groups was statistically significant (*P*<0.05). ALK mutations in lung adenocarcinoma were well predicted by the radiological features identified in this investigation. The AUC of the training set prediction model was 0.89 [95% CI: 0.84-0.95], and the AUC of the verification set was 0.79 [95% CI: 0.63-0.95], as shown in Fig. (**6a** and **b**).

### 3.3 Establishment, Evaluation and Verification of Radiological Nomogram

**Fig. (7):**
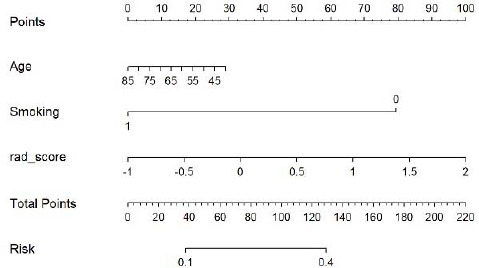
Radiomics nomogram for predicting ALK mutation in lung adenocarcinoma. Smoking: 1 indicated a history of smoking, while 0 indicated no smoking history. Based on a patient's age, smoking history, and radiomics score, a score was assigned and projected vertically onto the 'Points' line to obtain the indicator score. The scores of all indicators were then summed to obtain the total score, which was marked on the 'Total Points' line. A vertical line was projected from the total score to the 'Risk Probability' axis to determine the patient's risk probability of ALK mutation.

**Fig. (8):**
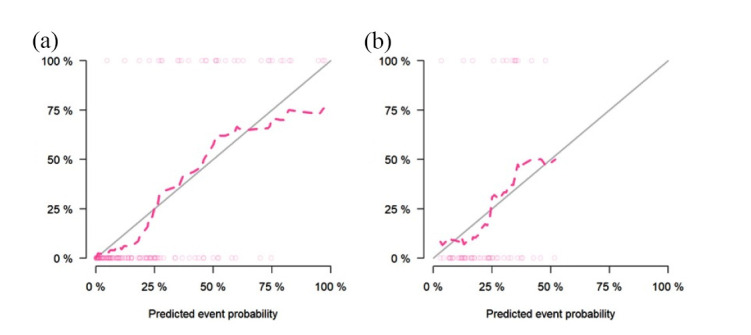
Calibration curves of the radiomics nomogram in the training set (**a**) and the validation set (**b**). The calibration curves assessed the agreement between the predicted ALK mutation status and the pathological results. The diagonal line represented perfect calibration, while the dashed line represented the performance of the nomogram. Closer proximity of the dashed line to the diagonal line indicated better calibration and prediction accuracy.

Table **2** suggests the clinical factors significantly associated with ALK mutation in lung adenocarcinoma, including age, TNM stage, smoking history and metastasis (*P* < 0.1). The results of multivariate logistic regression analysis showed that age, smoking history and radiomics score were independent indicators of ALK mutation in lung adenocarcinoma (Table **3**). Combining these factors, the radiomics nomogram was established (Fig. **7**). In the training and validation sets, the nomogram's AUCs were 0.91 and 0.80, respectively. The calibration curve showed that the actual nomogram probability and the forecast probability were fairly consistent (Fig. **8a, b**). The Hosmer-Lemeshow test revealed no statistically significant differences in calibration between the training dataset (*P* = 0.84) and the validation dataset (*P* = 0.36). Fig. (**9**) shows the DCA of the radiomics nomogram, which indicates that the radiomics nomogram performs better than the clinical model when the threshold probability falls between 0.1 and 1.0; the optimal threshold is 0.3.

### 3.4 The Prediction Effect of the Radiology Characteristic Model and the Radiology Nomogram

As shown in Table **4** and Fig. (**10a,b**), the AUC value of the radiomics nomogram in the training set was 0.91[95% CI: 0.86-0.96], with an accuracy of 83%, a sensitivity of 59% and a specificity of 96%; in the validation set, the AUC value was 0.80[95% CI: 0.63-0.97], with an accuracy of 81%, a sensitivity of 55% and a specificity of 95%. This suggests that the ALK mutation in lung adenocarcinoma has a good predictive ability using the radiomics nomogram developed in this work. The radiomics nomogram and radiomics signature did not differ significantly in terms of AUC (*P* = 0.1563 and 0.7725 in training and validation datasets, respectively). The radiomics nomogram's recognition performance (AUC = 0.80) outperformed the radiomics signature (AUC = 0.79). Both the radiomics signature and the radiomics nomogram outperformed the clinical models (radiomics nomogram *vs*. clinical model, *P* < 0.001 in the training set and *P* < 0.0337 in the validation set; radiomics signature *vs*. clinical model, *P* < 0.001 in the training set and *P* < 0.0150 in the validation set).

## 4 DISCUSSION

Previous imaging studies on ALK mutations have primarily focused on clinical characteristics and conventional imaging features. ALK Mut lung cancer is commonly associated with younger age, female gender, and non-smoking status. On CT, these tumors often appear solid and exhibit a tendency for lymph node metastasis [7-9, 24, 25]. Our study corroborated these findings, demonstrating that ALK Mut lung cancer frequently occurs in younger, non-smoking patients. Additionally, we observed a higher prevalence of advanced disease (stages III and IV) and a greater propensity for intrathoracic and distant organ metastasis in ALK Mut cases. Both ALK Mut and ALK WT were predominantly located in the periphery, with a female predominance. The ALK Mut group exhibited a slightly larger lesion size compared to the ALK WT group. While our study was consistent with previous reports regarding age, smoking history, and metastasis, we did not find significant differences in gender, maximum diameter, or location between the two groups.

Radiomics, a burgeoning field of research, utilizes computers to extract high-throughput quantitative features from medical images. These features capture tumor heterogeneity and facilitate the development of robust and practical predictive models. Radiomics offers enhanced objectivity and provides a comprehensive representation of tumor characteristics. Prior studies have demonstrated the potential of radiomics in predicting ALK mutation status in lung cancer [20-22, 26]. Yoon *et al*. [26] investigated the utility of CT radiomics in predicting ALK gene mutations. Their findings revealed that quantitative CT features extracted from local regions were significantly associated with the presence of ALK fusion genes. Song *et al*. [21] conducted a retrospective analysis of CT images from 335 lung adenocarcinoma patients. To predict ALK mutations, they used a machine learning model that included radiomics, clinical, and traditional CT data. The radiomics model's prediction performance in the training set was considerably enhanced by the addition of clinical and traditional CT features (*P* = 0.01). However, this improvement was not observed in the validation set (*P* = 0.29). As demonstrated in previous studies, a combined model that incorporates CT image-derived radiomic features and clinical attributes has been shown to effectively differentiate between ALK and EGFR mutation statuses in lung adenocarcinoma. Alternatively, CT image-based radiomics alone can also serve as a valuable tool for discrimination [20, 27].

Heterogeneity in tumor growth can lead to challenges in automatic segmentation when the grayscale intensity difference between the lesion and background is minimal. Manual segmentation ensures the accuracy of the region of interest (ROI) and remains the gold standard for clinical segmentation. Our study utilized manual segmentation to extract and screen texture features from patients' chest-enhanced CT images. Subsequently, an enhanced CT radiomics model was developed and validated to predict ALK mutations in lung adenocarcinoma. The model demonstrated significant value in predicting ALK mutations. Among the 19 features contributing to the model's prediction, wavelet features exhibited the highest predictive power. Our study also evaluated the predictive ability of the radiomics nomogram incorporating radiomics features and general clinical features. The radiomics nomogram outperformed the clinical model (AUC = 0.66) in the validation set, achieving an AUC of 0.80. However, in contrast to the radiomics prediction model, this improvement was not statistically significant (AUC = 0.79). Despite the lack of statistically significant improvement over the radiomics prediction model, the radiomics nomogram offers a user-friendly tool for personalized prediction of ALK Mut lung adenocarcinoma. It integrates both radiomics features and general clinical predictors, providing a comprehensive and easy-to-use approach. The radiomics nomogram model demonstrated a sensitivity of 55% and specificity of 95% in the validation set. While the sensitivity indicates a potential risk of missed diagnoses in identifying ALK mutation-positive patients, we believe the relatively small sample size, coupled with the 3-5% disease incidence for ALK mutations, may contribute to this limitation. Despite this, the high specificity underscores the model's effectiveness in accurately identifying negative cases.

Enhanced CT scans provide more comprehensive information compared to plain CT, including details on lesion blood supply and tumor heterogeneity. However, enhanced CT is susceptible to factors such as the contrast agent and operator variability, which can introduce bias into the results [28]. In our study, we carefully screened patient imaging data, excluding poor-quality cases to minimize interference, and standardized contrast agent protocols to reduce operator-related variability. Ma *et al*. [22] investigated the relationship between radiomics features of solid lung adenocarcinoma and ALK mutations. They performed ROI delineation and feature extraction on both plain and enhanced CT images of the patients' chests, and subsequently developed plain scan models and enhanced scan models. Their findings demonstrated that the enhanced CT radiomics features exhibited superior predictive performance for ALK mutations in solid lung adenocarcinoma compared to a plain CT classifier. In lung radiomics studies, researchers typically utilize either arterial or venous phase images for ROI delineation and feature extraction. However, given the continuous enhancement process of lung cancer during enhanced CT scanning, relying solely on one phase may result in the loss of valuable tumor lesion information present in the other phase. This study conducted ROI delineation and feature extraction on both the arterial and venous phases of patients' chest-enhanced CT images. These radiomics features were then used to create a CT prediction model. In the training and validation sets, the model's AUCs were 0.89 and 0.79, respectively. According to these findings, the enhanced CT prediction model that makes use of radiomics features performs admirably when it comes to identifying the presence of an ALK mutation in lung adenocarcinoma. In addition to the current research focus, many studies have concentrated on establishing imaging omics models for intratumoral and peritumoral features, as well as on the use of 18F-FDG PET-CT [29, 30].

The following are some of the study's limitations: 1) Sample size and study design: The relatively small sample size and single-center nature of the study may have limited the findings' external validity and generalizability. Future research should include multi-center studies with larger and more diverse populations to improve generalizability. 2) Scope of features: The study focused solely on CT radiomics features and general clinical features, excluding other potentially valuable predictors such as traditional imaging features, pathological classification, and laboratory indicators. Further exploration is needed to incorporate additional predictors (*e.g*., MRI, pathology, lab results) into a more comprehensive model, using multi-modal data to enhance predictive performance. 3) Lesion delineation: While manual segmentation provides more accurate radiological features, it requires significant time and effort. Additionally, manual delineation of the lesion on a single cross-sectional area of the enhanced CT image may introduce variability and subjectivity. Comparative verification using plain CT images and automatic segmentation algorithms was not performed in this study. Given the advancements in automatic image segmentation technology, future research should focus on implementing automated segmentation algorithms to reduce time and labor, while maintaining or even improving the accuracy of feature extraction. Moreover, incorporating multi-slice CT images and validating with other imaging modalities could further enhance accuracy.

## CONCLUSION

In conclusion, the established and validated prediction model utilizing enhanced CT radiomics features exhibits promising performance in predicting ALK gene mutations in lung adenocarcinoma. The model demonstrates the ability to offer an innovative, non-invasive approach to assist clinicians in making informed decisions regarding treatment strategies. However, this model warrants further exploration and validation in larger, multicenter studies to confirm its clinical utility and generalizability. Expanding the research to include a broader patient population will be essential to assess its robustness across different demographics and healthcare settings. Moreover, future studies could focus on enhancing the model by incorporating additional clinical and radiological factors, such as patient demographics, tumor heterogeneity, and molecular markers, to improve its predictive accuracy and reliability. Ultimately, refining this model could facilitate personalized medicine, leading to more precise and effective treatment options for lung adenocarcinoma patients, thereby improving patient outcomes and quality of life.

## Figures and Tables

**Fig. (1):**
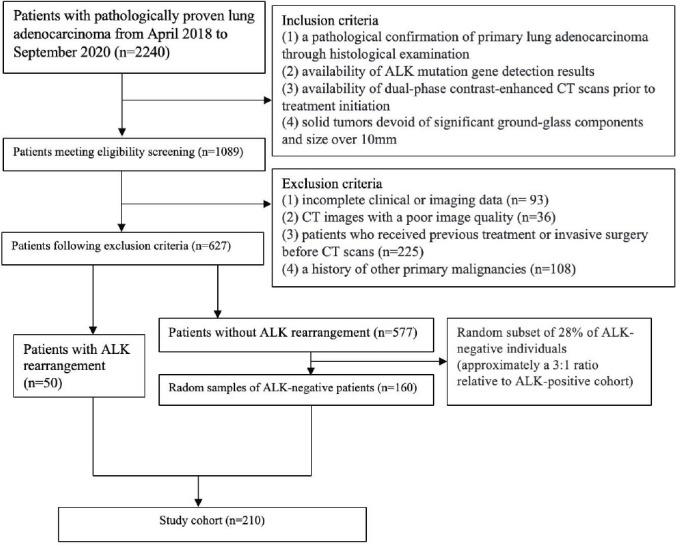
The flowchart of patient selection.

**Fig. (2):**
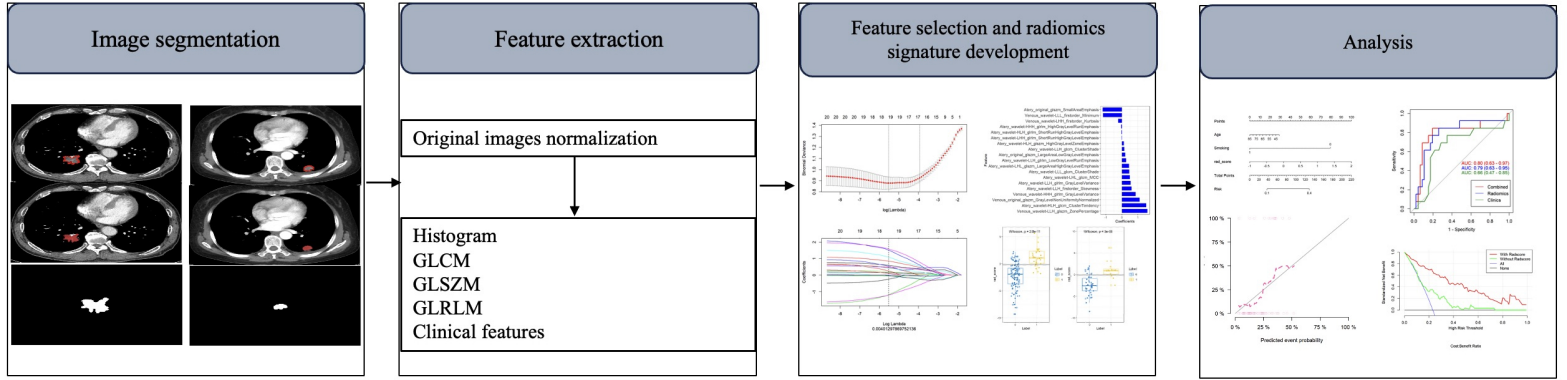
Workflow of the radiomics implementation process in the study.

**Fig. (5):**
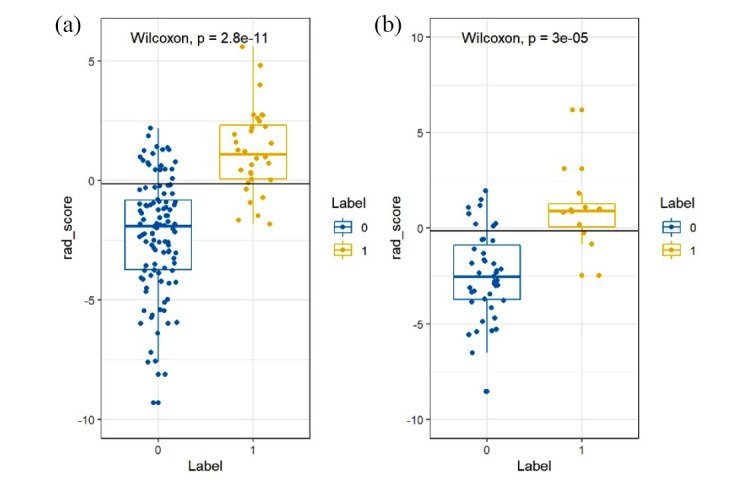
Radiomics scores of patients in the training set (**a**) and validation set (**b**). ALK mutation-negative was represented by 0, while ALK mutation-positive was represented by 1. The Wilcoxon test revealed statistically significant differences between the two groups, with *P-value*s less than 0.05.

**Fig. (6):**
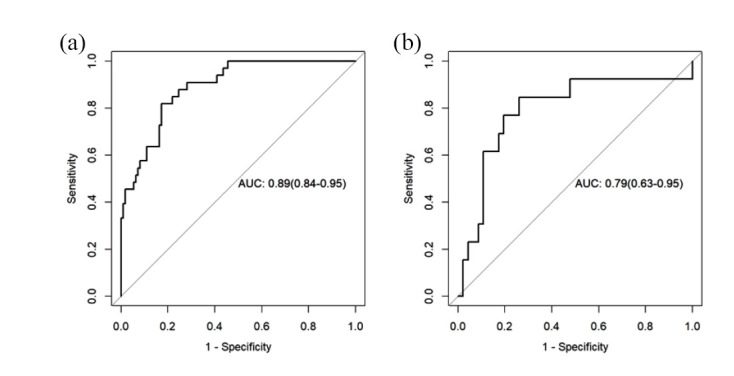
ROC curves of radiomics prediction model on training set (**a**) and validation set (**b**). The AUC for the training set was 0.89, and the AUC for the validation set was 0.79.

**Fig. (9):**
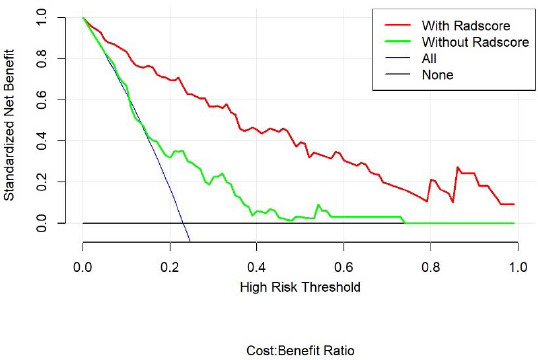
Decision curve analysis (DCA) of the radiomics nomogram. The y-axis represented net benefit, and the x-axis represented the threshold probability. The DCA demonstrated that utilizing the radiomics nomogram to predict ALK mutation status provided greater net benefit compared to treating all or none of the patients when the threshold probability ranged from 0.1 to 1.0. The optimal threshold was 0.3. This was illustrated by the green curve representing the clinical model, the blue line shows that all patients have ALK mutations, and the black line shows that no patients have ALK mutations.

**Fig. (10):**
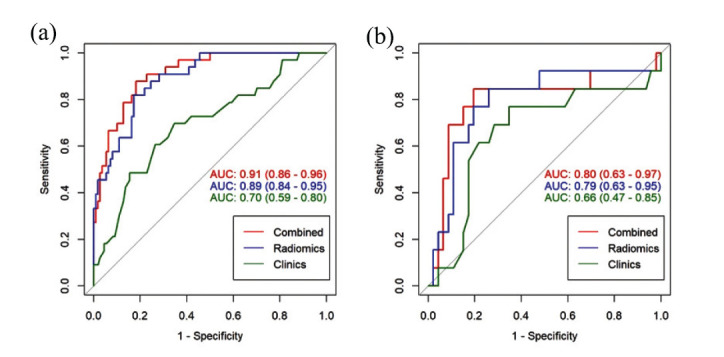
ROC curves of the radiomics nomogram, radiomics signature, and clinical model on the training set (**a**) and validation set (**b**). In the validation set (**b**), the radiomics nomogram (red line, AUC = 0.80) exhibited slightly better predictive performance compared to the radiomics signature (blue line, AUC = 0.79). In the training set (**a**), the radiomics nomogram (red line, AUC = 0.91) demonstrated superior predictive performance to both the clinical model (green line, AUC = 0.70) and the radiomics signature (blue line, AUC = 0.89).

**Table 1:** Comparison of clinical characteristics of ALK mutation-positive (ALK Mut) and ALK mutation-negative (ALK WT) patients with lung adenocarcinoma.

Characteristics	-	ALK Mut (n = 50)	ALK WT (n = 160)	*P-value*	t/*x*^2^-value
Age(years)		56.3±10.2	61.3±8.8	0.001	3.378
Sex	Male	18(36.0%)	70(43.7%)	0.332	0.940
	Female	32(64.0%)	90(56.3%)		
Smoking	Yes	9(18.0%)	54(33.7%)	0.034	4.500
	No	41(82.0%)	106(66.3%)		
TMN stage	Early (I, II)	19(38.0%)	91(56.8%)	0.020	5.441
	Advanced (III, IV)	31(62.0%)	69(43.2%)		
Metastatic	Yes	35(70.0%)	77(48.2%)	0.007	7.324
	No	15(30.0%)	83(51.8%)		
Diameter (cm)		3.2±1.5	3.1±1.8	0.613	-0.506
Location	Central	10(20.0%)	22(13.7%)	0.283	1.152
	Peripheral	40(80.0%)	138(86.3%)		

**Note:** Data are presented as the number of patients (%), or mean ± standard deviation. Pearson's Chi-squared test and t-test were used for statistical comparisons.

**Table 2:** Univariable logistic regression analysis of factors associated with ALK mutation in lung adenocarcinoma.

-	**Univariable Analysis**		-
Variable	OR	95% CI	*P-value*
Age	0.93	0.89-0.98	0.0027
TNM stage	2.58	1.16-6.02	0.0226
Smoking history	0.39	0.14-0.97	0.0554
Metastatic status	1.56	1.01-2.43	0.0463

**Abbrevaitions:** OR: odd ratio; CI: confidence interval.

**Table 3:** Multivariate logistic regression analysis of factors associated with ALK mutation in lung adenocarcinoma.

Multivariate Analysis			
Variable	OR	95% CI	*P-value*
Age	0.95	0.90-1.01	0.0930
Smoking history	0.26	0.07-0.89	0.0420
Radiomics score	2.67	1.89-4.15	<0.0001

**Table 4:** Performance of the radiomics nomogram, radiomics signature, and clinical model in predicting ALK mutation status in the training and testing sets.

Variable	-	Accuracy	Sensitivity	Specificity	PPV	NPV	AUC (95% CI)
Clinical model	Training	0.66	0.38	0.88	0.70	0.65	0.70(0.59-0.80)
	Validation	0.66	0.36	0.88	0.69	0.65	0.66(0.47-0.85)
Radiomics signature	Training	0.83	0.83	0.82	0.94	0.59	0.89(0.84-0.95)
	Validation	0.76	0.74	0.85	0.94	0.48	0.79(0.63-0.95)
Radiomics nomogram	Training	0.83	0.59	0.96	0.88	0.82	0.91(0.86-0.96)
	Validation	0.81	0.55	0.95	0.85	0.80	0.80(0.63-0.97)

Note: PPV, positive predictive value; NPV, negative predictive value.

## Data Availability

All the data and supporting information are provided within the article.

## LIST OF ABBREVIATIONS

ALK: Anaplastic Lymphoma Kinase
EGFR: Epidermal Growth Factor Receptor
CT: Computed Tomography
ROI: Region of Interest

## STANDARDS OF REPORTING

STROBE guidelines were followed.
